# The Effects of the WNT-Signaling Modulators BIO and PKF118-310 on the Chondrogenic Differentiation of Human Mesenchymal Stem Cells

**DOI:** 10.3390/ijms19020561

**Published:** 2018-02-13

**Authors:** Xiaobin Huang, Leilei Zhong, Jan Hendriks, Janine N. Post, Marcel Karperien

**Affiliations:** Developmental BioEngineering, MIRA Institute for Biomedical Technology and Technical Medicine, University of Twente, Enschede 7500 AE, The Netherlands; x.huang-1@utwente.nl (X.H.); zhongleilei8@gmail.com (L.Z.); j.hendriks@utwente.nl (J.H.); j.n.post@utwente.nl (J.N.P.)

**Keywords:** hMSC, WNT, chondrogenesis, cartilage, tissue engineering

## Abstract

Mesenchymal stem cells (MSCs) are multipotent cells, mainly from bone marrow, and an ideal source of cells in bone and cartilage tissue engineering. A study of the chondrogenic differentiation of MSCs is of particular interest for MSCs-based cartilage regeneration. In this study, we aimed to optimize the conditions for the chrondogenic differentiation of MSCs by regulating WNT signaling using the small molecule WNT inhibitor PKF118-310 and activator BIO. Human mesenchymal stem cells (hMSCs) were isolated from bone marrow aspirates and cultured in hMSCs proliferation medium. Pellet culture was subsequently established for three-dimensional chondrogenic differentiation of 5 weeks. WNT signaling was increased by the small molecule glycogen synthase kinase-3 inhibitor 6-bromoindirubin-3-oxim (BIO) and decreased by the WNT inhibitor PKF118-310 (PKF). The effects of BIO and PKF on the chondrogenesis of hMSCs was examined by real-time PCR, histological methods, and ELISA. We found that activation of canonical WNT-signaling by BIO significantly downregulated the expression of cartilage-specific genes *SOX9*, *COL2A1,* and *ACAN*, and matrix metalloproteinase genes *MMP1/3/9/13,* but increased *ADAMTS 4/5*. Inhibition of WNT signaling by PKF increased the expression of *SOX9*, *COL2A1*, *ACAN*, and *MMP9,* but decreased *MMP13* and *ADAMTS4/5*. In addition, a high level of WNT signaling induced the expression of hypertrophic markers *COL10A1, ALPL*, and *RUNX2,* the dedifferentiation marker *COL1A1*, and glycolysis genes *GULT1* and *PGK1*. Deposition of glycosaminoglycan (GAG) and collagen type II in the pellet matrix was significantly lost in the BIO-treated group and increased in the PKF-treated group. The protein level of COL10A1 was also highly induced in the BIO group. Interestingly, BIO decreased the number of apoptotic cells while PKF significantly induced apoptosis during chondrogenesis. The natural WNT antagonist DKK1 and the protein level of MMP1 in the pellet culture medium were decreased after PKF treatment. All of these chondrogenic effects appeared to be mediated through the canonical WNT signaling pathway, since the target gene *Axin2* and other WNT members, such as *TCF4* and *β-catenin*, were upregulated by BIO and downregulated by PKF, respectively, and BIO induced nuclear translocation of β-catenin while PKF inhibited β-catenin translocation into the nucleus. We concluded that addition of BIO to a chondrogenic medium of hMSCs resulted in a loss of cartilage formation, while PKF induced chondrogenic differentiation and cartilage matrix deposition and inhibited hypertrophic differentiation. However, BIO promoted cell survival by inhibiting apoptosis while PKF induced cell apoptosis. This result indicates that either an overexpression or overinhibition of WNT signaling to some extent causes harmful effects on chondrogenic differentiation. Cartilage tissue engineering could benefit from the adjustment of the critical level of WNT signaling during chondrogenesis of hMSC.

## 1. Introduction

The treatment of injuries to hyaline cartilage is considered a challenge due to the limited ability of self-repair of articular cartilage. Although much research effort has been made to improve the regeneration of articular cartilage, there is no curative treatment available yet for rebuilding native articular cartilage. Human mesenchymal stem cells (hMSCs) are multipotent cells mainly from the bone marrow. They can be expanded in vitro and be differentiated into the osteogenic, chondrogenic, and adipogenic lineages [1], and are considered suitable candidates for the promotion of tissue regeneration [2,3]. A study of cartilage differentiation of MSCs would be of particular interest for the cell-based treatment of cartilage defects, and optimization of MSCs chondrogenesis is of particular importance. Since WNT signaling is indicated to be involved in the chondrogenic differentiation of hMSCs, the chondrogenic process can be optimized by regulating WNT signaling.

WNT signaling plays an extremely important role in the regulation of cell proliferation and differentiation in embryonic and cartilage development [4,5]. When inactive, β-catenin is phosphorylated by glycogen synthase kinase (GSK) 3β. The phosphorylated β-catenin undergoes subsequent ubiquinitylation and proteasomal degradation. Conversely, in an active state, WNT ligands bind to frizzled receptors and their co-receptor of low-density lipoprotein receptor-related proteins (LRP) 5 or 6. This activates disheveled (DSH) that subsequently inhibits GSK3β activity. As a result, β-catenin will not undergo degradation, and will accumulate then translocate into the nucleus. In the nucleus, it interacts with transcription factors of the T-cell specific transcription factor/lymphoid enhancer-binding factor (TCF/LEF) family to initiate the transcription of target genes [6,7]. GSK3β is a key molecular regulator of the WNT pathway, since its inhibition initiates the signaling pathway [8,9,10].

Several small molecules that regulate WNT signaling have been reported [11]. In our study, we focus on two important regulators: BIO and PKF118-310 (PKF). BIO activates canonical WNT signaling in human chondrocytes by blocking GSK-3β [12]. The small molecule PKF inhibits WNT signaling by interfering with the binding of β-catenin to the transcription factor TCF4 [13,14,15].

The potential effects of small molecules that activate/inhibit WNT/β-catenin signaling on cell culture have been demonstrated. Some studies have reported that BIO could promote cell growth [16,17,18,19] and maintain the pluripotency of an embryonic stem cell when supplemented in the culture medium [19,20]. A recent in vitro study has shown that the addition of 0.01 µM BIO promotes the chondrogenesis of mouse MSCs that are bone-marrow-derived [21]. A previous study by our group has suggested that small molecules that have inhibited WNT signaling effectively prevent IL1β- and TNFα-induced cartilage degradation by blocking MMP expression and activity [22]. PKF, an inhibitor of WNT/β-catenin signaling, inhibits the growth of hepatocellular carcinoma (HCC) cells as well as targets breast tumor-initiating cells in a mouse model of breast cancer in vitro and in vivo [14,23,24].

Even though many studies focus on small inhibitors or activators of WNT/β-catenin signaling in cell research, there is no report about the effect of an inhibitor/activator on hMSCs chondrogenesis in vitro. The aim of this study was to assess the potential effects of small molecules on chondrogenic-differentiating hMSCs with continuous inhibition/activation of canonical WNT signaling using a three-dimensional (3-D) pellet culture system.

## 2. Results

### 2.1. The Efficacy and Specificity of BIO and PKF on the TCF/LEF Reporter

**Figure 1:**
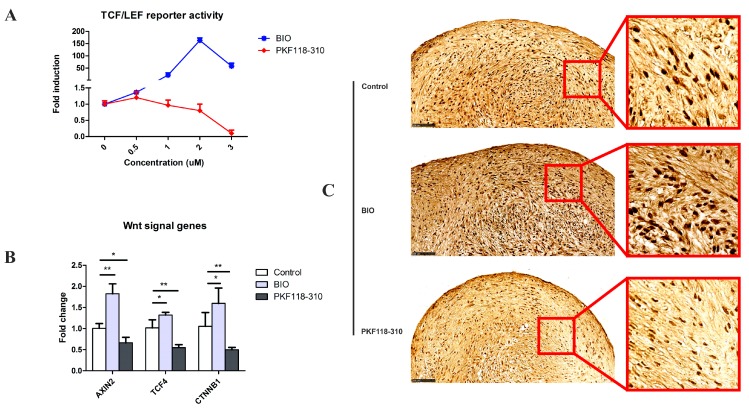
The efficacy and specificity of BIO and PKF on TCF/LEF reporter and hMSCs. (**A**) TCF/LEF reporter assay. To measure TCF/LEF reporter activity, HEK293T cells were infected with a lentiviral TCF/LEF reporter construct and stimulated with different concentrations of BIO or PKF for 24 hours (h), and luciferase activity was measured using the Dual-Glo luciferase assay kit. Data was expressed as fold change compared to control. (**B**) The expression of WNT signaling responsive genes in hMSCs. * *p*-value (*p*) < 0.05; ** *p* < 0.01. The error bar represents the Standard Deviation (SD). (**C**) Immunohistochemistry of β-catenin in hMSCs pellets. The expression of β-catenin was detected by rabbit anti-human β-catenin antibody, followed by incubation with the biotinylated secondary anti-rabbit antibody and horseradish peroxidase (HRP)-Streptavidin using a DAB (3′-Diaminobenzidine) substrate kit. Images were taken by using a Nanozoomer. Scale bar = 100 µm.

In order to test the efficacy and specificity of BIO and PKF in terms of activating/blocking WNT/β-catenin signaling, dose-response experiments were performed in HEK293t cells transfected with the TCF/LEF reporter. The cells were cultured in the medium with or without BIO/PKF. We found a dose-dependent increase in reporter activity when the cells were treated with BIO. Maximal activation was found at 2 µM. PKF treatment resulted in a 10-fold decrease in reporter activity at a concentration of 3 µM (Figure 1A). On the basis of this result, we adopted the optimal concentration of BIO and PKF of 2 and 3 µM, respectively, in all subsequent experiments.

To verify whether the WNT signaling pathway is activated or inhibited by small molecules in hMSCs pellets, the effects of BIO and PKF118-130 on the expression of the WNT target gene *AXIN2,* and the WNT-related genes *TCF4* and *CTNNB1* (gene encoding β-catenin protein), were measured by qPCR. The expression and nuclear translocation of β-catenin was detected by immunohistochemistry (IHC). We observed that BIO significantly induced mRNA expression of the canonical WNT target gene *AXIN2* and the WNT signal related genes *TCF4* and *CTNNB1*, while PKF effectively inhibited canonical WNT signaling (Figure 1B). IHC results showed that the cells had a basic β-catenin expression level. It was observed that BIO strongly enhanced the β-catenin expression and promoted its nucleus translocation, while PKF decreased the expression and inhibited nuclear translocation (Figure 1C). All of these results suggested that canonical WNT signaling was efficiently activated by BIO and inhibited by PKF.

### 2.2. The Effects of Activation and Inhibition of Canonical WNT Signaling on Gene Expression during hMSCs Chondrogenesis

**Figure 2:**
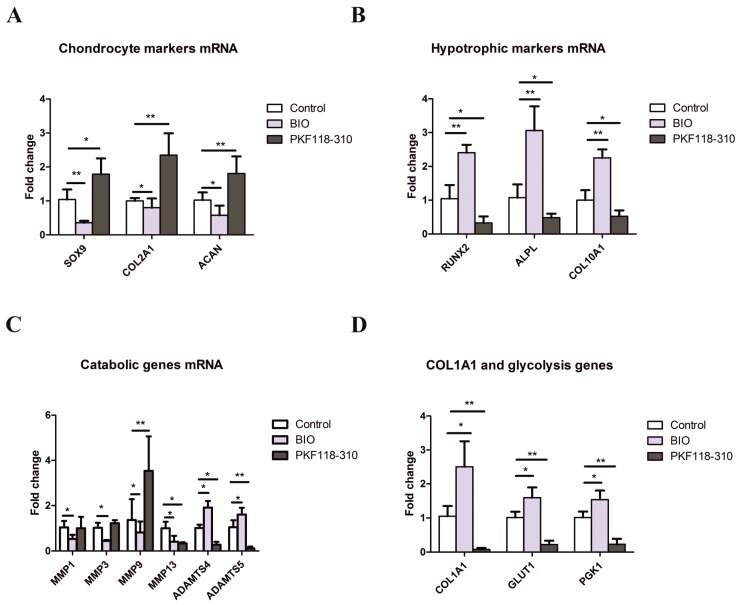
Gene expression in hMSCs pellets after activating/inhibiting canonical WNT signaling. The expression of (**A**) chondrocyte markers; (**B**) hypertrophic markers; (**C**) catabolic genes; and (**D**) *COL1A1* and glycolysis genes. hMSCs pellets were cultured in chondrogenic differentiation medium with supplementation of 2 µM BIO or 3 µM PKF for 5 weeks. After 5 weeks, RNA samples were extracted from pellets and the expression of specific genes was analyzed by qPCR. Relative expression levels of different genes were normalized using RPL13A. Data are presented as an average of three hMSCs donors ± SD. * *p* < 0.05, ** *p* < 0.01. The error bar represents the Standard Deviation (SD).

To investigate the effects of canonical WNT signaling on chondrogenesis of hMSCs, the gene expression of chondrocyte markers was measured by qPCR after 5 weeks of pellet culture in the absence or presence of BIO or PKF. The expression of typical cartilage markers, such as *SOX9*, *COL2A1*, and *ACAN,* was significantly downregulated by BIO. However, inhibition of WNT signaling by PKF resulted in upregulation of *SOX9*, *COL2A1*, and *ACAN* (Figure 2A).

In a previous study, we suggested that a high level of WNT signaling induces the chondrogenic differentiating hMSCs to undergo hypertrophic differentiation, and that addition of the WNT natural antagonists DKK1 and FRZB prevents the hypertrophy of hMSCs during chondrogenesis [25]. We, therefore, explored the effect of high versus low WNT signaling on the expression of hypertrophic markers. In line with our previous study, a high level of canonical WNT signaling induced by BIO significantly increased the expression of *RUNX2*, *ALPL*, and *COL10A1*. In PKF-treated cells, which displayed a low total non-detectable WNT signal, the expression of these hypertrophic genes was significantly decreased (Figure 2B).

We then examined the effect of canonical WNT signaling on the expression of catabolic genes *MMPs* and *ADAMTS 4/5*. Interestingly, treatment of BIO significantly decreased the expression of *MMP 1/3/9/13*, while it increased aggrecanase *ADAMTS 4/5* expression. However, *MMP9* increased in the PKF-treated group while *MMP13* and *ADAMTS 4/5* strongly decreased. *MMP1*/*3* did not show a significant change (Figure 2C).

The dedifferentiation marker *COL1A1* was also measured by qPCR. As Figure 2D shows, BIO-induced high WNT signaling greatly increased the expression of *COL1A1*, while PKF-inhibited WNT signaling dramatically decreased *COL1A1* expression. In addition, we also observed that the two key glycolysis genes *GULT1* and *PGK1* were highly expressed in the BIO group and less highly expressed in the PKF group (Figure 2D).

### 2.3. High Levels of Canonical WNT Signaling Inhibited Cartilage Formation

**Figure 3:**
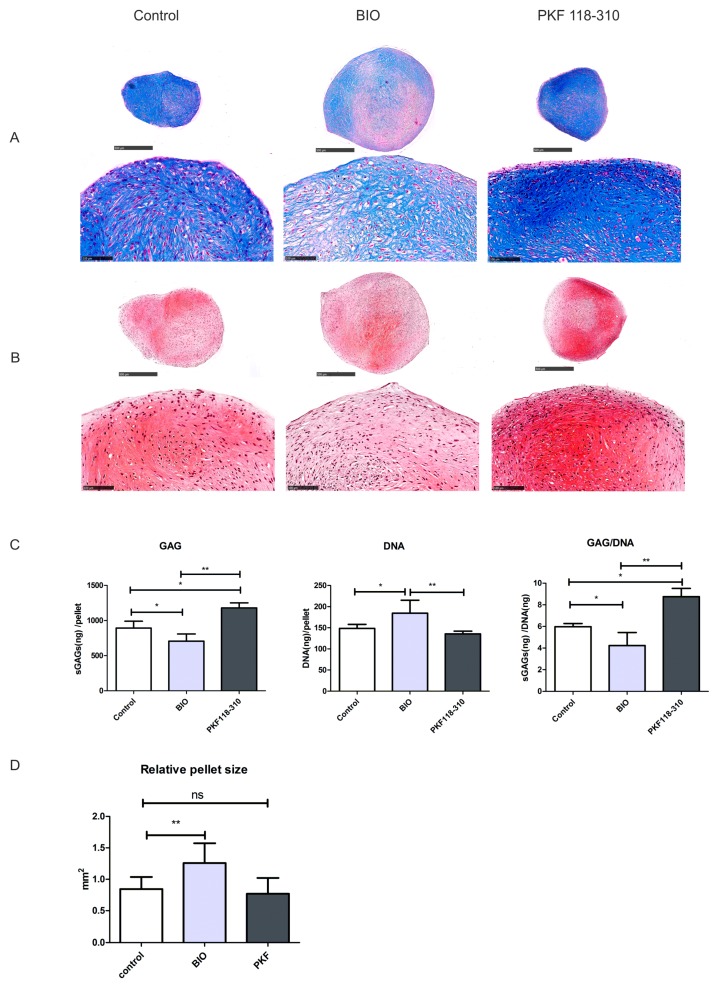
Activation of the canonical WNT signaling pathway inhibits cartilage formation. The effects of small molecules on cartilage formation and pellet size after 5 weeks of culture. (**A**) Alcian blue staining was performed to examine the deposition of sulfated glycosaminoglycans (GAGs) in midsagittal paraffin sections of hMSCs pellets; nuclei were counterstained with nuclear fast red. The upper panel indicates the overview of each pellet, scale bar = 500 μm; the lower panel indicates magnified pictures, scale bar = 100 μm; (**B**) Safranin O staining; nuclei were counterstained with hematoxylin. The upper panel indicates the overview of each pellet, scale bar = 500; the lower panel indicates magnified pictures, scale bar = 100 μm; (**C**) GAG and DNA production in pellet; (**D**) Quantification of the average size of the pellets. * *p* < 0.05, ** *p* < 0.01. Error bars represent SD.

**Figure 4:**
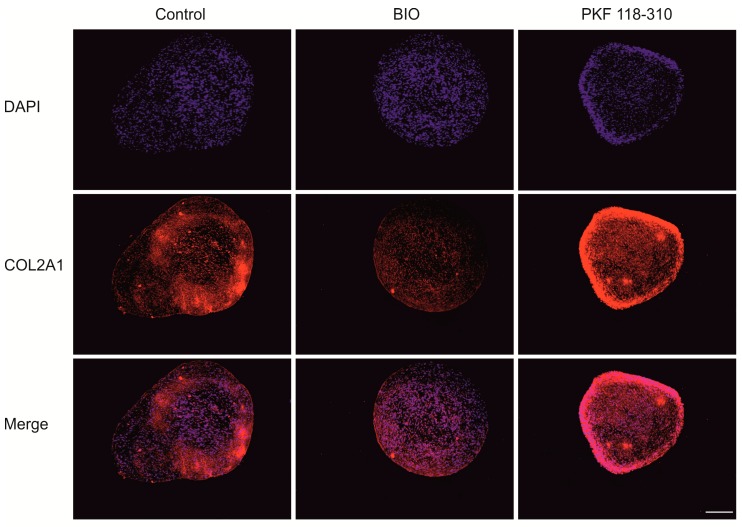
Activation of canonical WNT signaling prevents collagen type 2 production, while inhibition of canonical WNT signaling enhances collagen type 2 production. The expression of type II collagen was visualized by immunofluorescence in midsagittal paraffin sections. The Alexa^®^Fluor 546-labelled second antibody was used to detect the positive type II collagen expression. The nucleus was counterstained by DAPI. Scale bar = 500 μm.

To study the contribution of canonical WNT signaling to cartilage matrix formation after 5 weeks of chondrogenesis, histological staining was performed. Alcian blue/Safranin O staining and a glycosaminoglycans (GAG) assay (Figure 3A–C) indicated that BIO treatment decreased cartilage formation, while PKF increased cartilage formation. Moreover, the addition of BIO increased both the total DNA synthesis and the average diameter of the pellets significantly. There were no significant differences between the DNA content and pellet size in the PKF and control groups (Figure 3C,D). In line with the GAG staining and the chemical assay, immunofluorescence indicated that BIO slightly downregulated collagen type II expression, while PKF promoted its expression (Figure 4).

### 2.4. BIO Significantly Decreased the Number of Apoptotic Cells, While PKF Induced hMSCs Apoptosis in Pellets

**Figure 5:**
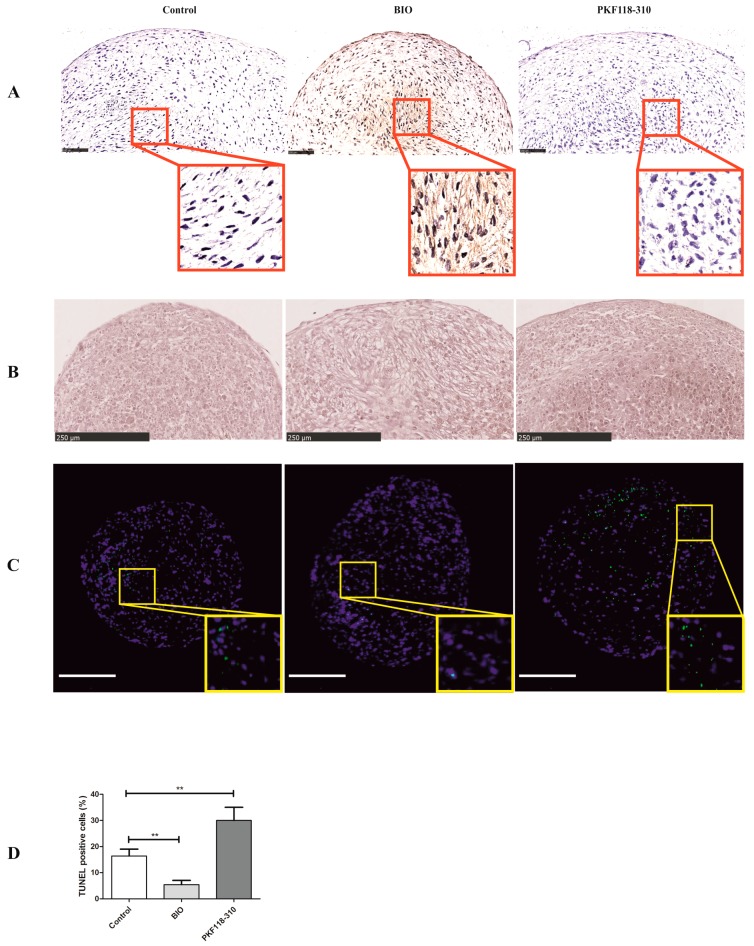
The effects of BIO or PKF on the hypertrophy, mineralization, and apoptosis of hMSCs pellets. (**A**) Immunohistochemistry of Collagen type X. The Collagen type X was detected by mouse anti-human Collagen type X antibody. Cell nuclei were counterstained with hematoxylin. Images were taken using a Nanozoomer. Scale bar = 100 µm; (**B**) Alizarin red staining for matrix mineralization. Scale bar = 250 µm; (**C**) TUNEL assay. Positive apoptosis cell (green) was stained using the DeadEnd Fluorometric TUNEL System. Nuclei were counterstained with Hoechst 33342 (blue). Scale bar = 500 µm; (**D**) Quantification of apoptotic cells. ** *p* < 0.01. Error bars represent SD.

It is evident from the qPCR result that BIO significantly induced the expression of hypertrophic markers. We assumed that the treatment of BIO might induce cells towards hypertrophic differentiation. In order to investigate hypertrophic differentiation, the typical hypertrophy marker type X collagen was detected by immunohistochemistry. Type X collagen could hardly be detected in either the control or the PKF group. However, BIO greatly induced the expression of COL10A1 (Figure 5A).

During the process of hypertrophic differentiation, cells will enlarge, terminally differentiate, and ultimately undergo mineralization. In order to further elucidate this process, Alizarin red S staining was performed to evaluate mineralization. No matrix mineralization was observed in any of the groups (Figure 5B).

Several members of the WNT family, such as WNT16, WNT7a, and TCF4, have been shown to be involved in the regulation of chondrocyte apoptosis [26,27,28]. In order to investigate if the alteration of WNT signaling by BIO/PKF will modulate cell apoptosis, a TUNEL assay was used to detect apoptotic cells. Apoptotic cells were highly present in the PKF group but barely present in the BIO treatment, as shown in Figure 5B. Compared to the control, the number of apoptotic cells was decreased in the BIO-treated group while it had increased in the PKF-treated group (Figure 5C).

### 2.5. The Protein Level of DKK1 and MMP1 Was Decreased in Pellet Culture Medium with PKF Treatment

**Figure 6:**
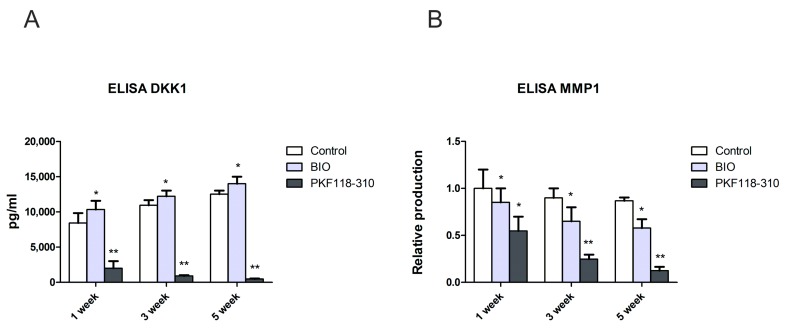
The protein concentration of DKK1 and MMP1 in the pellet culture medium. hMSCs pellets were treated with BIO or PKF for 5 weeks. The medium was collected in week 1, 3, and 5, respectively. The concentrations of DKK1 (**A**) and MMP1 (**B**) were measured by ELISA. * *p* < 0.05, ** *p* < 0.01. Error bars represent SD.

Previously, we found that DKK1, as a natural WNT antagonist, plays a vital role in the regulation of chondrogenic differentiation of hMSCs. In this study, we collected the medium and measured the concentration of endogenous DKK1 secreted from hMSC pellets after treatment with BIO or PKF by ELISA. After continuous stimulation for 5 weeks, BIO slightly increased the expression of DKK1. However, PKF dramatically decreased DKK1 expression to a very low level (Figure 6A). The relative protein level of MMP1 was also measured in the collected medium. The concentration of MMP1 gradually decreased over time in all the treatments. Both BIO and PKF decreased the MMP1 expression. Compared to BIO treatment, the PKF treatment inhibited the expression of MMP1 more effectively (Figure 6B).

## 3. Discussion

Cell-based cartilage regeneration often involves the use of chondrocytes or hMSCs. However, hMSCs chondrogenic differentiation is still unsatisfactory. An improvement in current cartilage tissue-engineering protocols requires more detailed insight into the molecular mechanisms that regulate the distinct steps of chondrogenic differentiation of hMSCs. We propose that targeting the canonical WNT signaling pathway to increase chondrogenic differentiation of MSCs would be a very important and a valuable strategy.

In the present study, we have attempted to investigate the effect of two small molecules, BIO and PKF, which have been described as positive and negative regulators of the WNT signaling pathway [12,13,14,15]. Our objective was to further improve the chondrogenic microenvironment by regulating canonical WNT signaling in chondrogenic differentiation medium. In addition, we aimed to recapitulate the dual role of WNT signaling in chondrogenically differentiating hMSCs.

We found that the addition of the canonical WNT activator BIO downregulated the expression of cartilage-specific genes and increased cartilage matrix degradation. In contrast, addition of the WNT inhibitor PKF upregulated the expression of cartilage markers and increased the cartilage matrix deposition. This is consistent with other findings showing that the expression pattern of three cartilage-specific genes are downregulated in cultures with 1 µM BIO treatment in mice MSCs cells, while PKF increased the cartilage markers in mouse fetal metatarsals [21,22].

The effects of BIO and PKF on chondrogenic differentiation are mediated through the canonical WNT signaling pathway, as both factors modulate the WNT pathway via β-catenin. BIO functions through preventing β-catenin breakdown through the inhibition of GSK3β, while PKF prevents the interaction of β-catenin with transcription factors, TCF/LEF. The observed effects of BIO and PKF are in line with the observations from transgenic animals in which β-Catenin is modulated [29,30]. We therefore conclude that our observations are mainly caused by the perturbation of WNT signaling, although the contribution of minor off-target effects cannot be excluded.

Mesenchymal stem cells show promising results in cell-based treatment for cartilage regeneration. Unfortunately, more and more studies are suggesting that the loss of the chondrogenic phenotype and expression of hypertrophic markers, including COL10A1, MMP13, and VEGF, are observed in chondrogenically differentiated hMSCs [31,32,33]. This raises problems for the clinical application of MSCs, since hypertrophy could eventually lead to ossification and apoptosis. WNT/β-catenin signaling in cartilage plays a dual role. Activity is essential for chondrocyte proliferation and maintenance of the phenotypic characteristics [34], but excessive activity increases chondrocyte hypertrophy and the expression of cartilage-degrading matrix metalloproteinases [35,36]. In this study, we found that PKF inhibited cell hypertrophy by inhibiting hypertrophic markers, while BIO induced the expression of hypertrophic markers. This is in line with our previous study, which showed that blocking WNT signaling by its antagonists DKK1 and FRZB decreases the hypertrophic differentiation of hMSCs [37]. Similarly, activation of WNT signaling by neutralization of DKK1 and FRZB promotes hypertrophy of chondrogenic differentiating hMSCs and subsequent matrix mineralization, while it decreases the expression of cartilage markers [25,37]. In this study, the measured DKK1 concentration by ELISA was extremely low in the PKF treatment, whichcould serve as a positive feedback of modulation for low WNT signaling as regulated by PKF.

MMPs are the main proteinases degrading collagens and proteoglycans in joint disease. Previous research by our group shows that, in human chondrocytes, WNT/β-catenin is a potent inhibitor of MMP expression in both basic and IL1β-treated conditions. The results also show that WNT3a decreased the mRNA expression of *MMP 1/3/13* in human MSCs [38,39]. Similar effects of high WNT level on MMPs were observed in this study. More specifically, BIO significantly decreased the expression of *MMP1/3/9/13* in hMSCs. The WNT inhibitor PKF was found to decrease *MMP13*, but increased the *MMP9* expression. Although MMP13 is one of the proteinases, it is also a typical hypertrophic marker. Its expression can be decreased by alternative pathways, such as the RUNX2- and FGF2-mediated pathways [40]. The MMP1 protein level dramatically decreased in the PKF-treated medium. We cannot explain the inconsistent expression of MMP1 between protein and gene level on the basis of our current knowledge. It may be that alternative pathways are involved in the regulation of MMP1 post-translational modification.

It has been shown that overexpression of β-catenin, using a β-catenin activator or in APC knockout mice, leads to the dedifferentiation of chondrocytes [41,42]. We found that a high level of WNT signaling induced by BIO significantly increased *COL1A1* expression, which further proves the role of WNT/β-catenin signaling in chondrocyte dedifferentiation. In addition, considering the metabolic ATP requirement of MSCs shifts from oxidative phosphorylation to predominantly glycolytic metabolism during chondogenic differentiation [43], two key glucolysis genes, *GLUT1* and *PGK1*, were detected in this study. Interestingly, their expression was elevated in BIO group but dramatically decreased in the PKF group, which may indicate that the glucolysis metabolism in PKF-treated cells was inhibited greatly.

Mineralization is the ultimate end stage of chondrocyte hypertrophy. To investigate whether BIO-treated chondrogenically differentiating MSCs also undergo matrix mineralization, Alizarin red S staining was adopted to observe the mineralization nodule. No mineralization was observed in either the control or the experimental group in this study, which suggests that the hypertrophic differentiation did not reach the end stage. In addition, it has been shown that chondrocyte hypertrophy does not have to be related to chondrocyte mineralization [44].

Apoptosis refers to the autonomous and orderly death of gene-controlled cells in order to maintain a stable internal environment, and is an active process involving the activation, expression, and regulation of a range of genes without inducing inflammation [45]. One study has shown that inhibition of β-catenin signaling in articular chondrocytes causes increased cell apoptosis in a transgenic mice model [46]. It has also been demonstrated that hMSCs alone or in co-culture with chondrocytes undergo apoptosis during chondrogenesis due to serum withdrawal and prolonged cell culture [47,48]. In our study, the apoptosis assay has shown that apoptotic cells were present in both control and experimental groups cultured in medium without serum. Many studies have reported BIO as an anti-apoptotic agent that inhibits apoptosis after serum deprivation [49,50]. We found that BIO significantly decreased the number of apoptotic cells, while PKF induced cell apoptosis.

Activated WNTβ-catenin signaling is well-known to stimulate cell proliferation in multiple cell cultures in vitro [16,17,18,51,52]. We observed that pellets with BIO treatment showed increased diameter and DNA numbers. Considering the anti-apoptotic effects and proliferation stimulating effects of BIO on cultured cells, it is no surprise that the pellets enlarged after BIO treatment.

**Figure 7:**
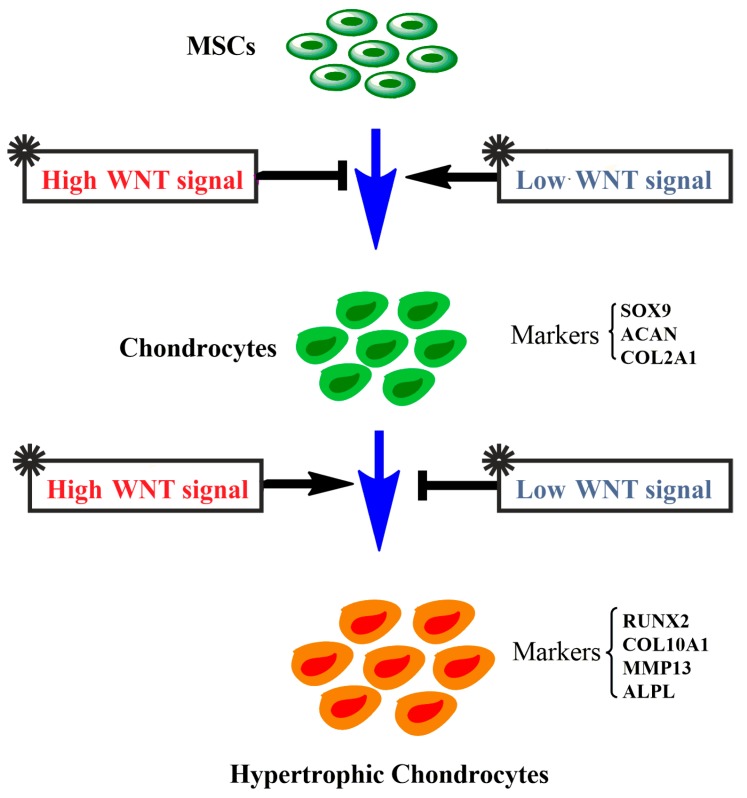
The schematic overview for the chondrogenic differentiation of hMSCs regulated by WNT signaling. High levels of WNT signaling inhibit the chondrogenic differentiation of hMSCs, evidenced by decreased *SOX9*, *ACAN*, and *COL2A1* expression, but promote the hypertrophy of chondrocytes, exemplified by the induction of expression of *RUNX2*, *COL10A1*, *MMP13*, and *ALPL*. In contrast, low levels of WNT signaling promote hMSC chondrogenic differentiation and inhibit chondrocyte hypertrophy.

Taken together, the chondrogenic differentiation of hMSCs, characterized by high expression of chondrocyte markers *SOX9*, *COL2A1*, and *ACAN*, was inhibited by BIO-induced high WNT signaling, while being promoted by the lower WNT activity after PKF treatment. Moreover, active canonical WNT signaling induced the expression of chondrocyte hypertrophic markers, such as *RUNX2*, *COL10A1*, and *ALPL*, while their expression was reduced by lowering the WNT activity (Figure 7). Our findings suggest that fine-tuning canonical WNT signals in the chondrogenic differentiation process of hMSCs could contribute to better cartilage tissue engineering.

## 4. Materials and Methods

### 4.1. Luciferase Assay

HEK293t cells were infected with Cignal^TM^ lentiviruses containing a TCF/LEF luciferase reporter construct (SABiosciences, Frederick, MD, USA) together with lentiviruses constitutively expressing Renilla luciferase (SABiosciences). HEK293t cells were seeded at 8000 cells/cm^2^ in 96-wells plates (Nunc international, Rochester, NY, USA) and cultured for 24 h in basic medium (DMEM supplemented with 10% FBS and 100 µ/mL penicillin and streptomycin). The cells were treated with BIO (Sigma Aldrich, St. Louis, MO, USA) or PKF118-310 (PKF) (Sigma Aldrich) for 24 h. After 24 h, the cells were lysed in Glo lysis buffer and luciferase activity was measured using a Dual-Glo luciferase assay kit (Promega, Madison, WI, USA).

### 4.2. Cell Culture and Expansion

The use of human bone marrow aspirates was approved by a local medical ethics committee (study protocol K06-002, 2 January 2006) and written informed consent by the donors was obtained. The aspirates were resuspended using a 20 Gauge needle and plated at a density of 0.5 million mononucleated cells/cm^2^. MSCs were selected by adherence in a proliferation medium (α-MEM supplemented with 10% fetal bovine serum, 1% l-glutamax, 0.2 mM ascorbic acid, 100 U/mL penicillin, 10 mg/mL streptomycin, and 1 ng/mL bFGF).

### 4.3. Pellet Culture and Chondrogenic Differentiation of hMSCs

To obtain pellet-cultures, 250,000 MSCs at passage three were seeded in 10 mL round-bottom tubes in chondrogenic differentiation medium (DMEM supplemented with 40 μg/mL proline, 50 µg/mL ITS-premix, 50 µg/mL AsAP, 100 µg /mL Sodium Pyruvate, 10 ng/mL TGFβ3, 10^−7^ M dexamethasone, 100 U penicillin/mL, and 100 μg/mL streptomycin) and centrifuged for 5 min at 500× *g*. Three groups were included in this experiment: hMSC pellets in chondrogenic differentiating medium as the control group, hMSC pellets with BIO treatment, and hMSC pellets with PKF treatment. BIO and PKF were used at a concentration of 2 and 3 µM, respectively. Three donors were used for each condition as biological triplicates. Cell pellets were grown at 37 °C in a humid atmosphere with 5% CO_2_. The medium was refreshed twice a week. The cell pellets were cultured for 5 weeks before analysis.

### 4.4. RNA Isolation and Quantitative PCR

RNA samples of cell pellets were isolated using TRIzol (Thermo Fisher Scientific, Waltham, MA, USA) according to the manufacturer’s protocol. The concentration and purity of the RNA samples were measured with the Nanodrop2000. The total RNA was reverse-transcribed into cDNA using the iScript cDNA Synthesis kit (Bio-Rad, Hercules, CA, USA). Quantitative PCR (qPCR) was performed as [53].

### 4.5. Alcian Blue/Safranin O and Alizarin Red Staining

Chondrogenically differentiated hMSC pellets were fixed with 10% buffered formalin for 15 minutes (min) and embedded in paraffin using routine procedures. Sections of 5 μm were cut using a microtome (Shandon, Waltham, MA, USA). Alcian blue/Safranin O staining was performed as described previously [54]. The sections were also stained with Alizarin red staining for mineralization as [37].

### 4.6. Immunofluorescent (IF) Staining for Collagen Type II

For the immunofluorescent staining of collagen type II, 5 µM sections were de-paraffinized in xylene and rehydrated with graded ethanols. The samples were pre-incubated with 5 μg/mL proteinase K (Sigma Aldrich) for 10 min at room temperature (RT) followed by 1 mg/mL hyaluronidase (Sigma Aldrich) for 40 min at 37 °C. They were blocked in 5% BSA in PBS for 1 h, after which they were incubated overnight at 4° with rabbit anti-human collagen II antibody (Abcam, Cambridge, UK), which was diluted 1:100 in 5% BSA in PBS. The cells were rinsed three times with PBST, 5 min/time. Then, Alexa^®^Fluor 546-labelled goat anti-rabbit antibody in 5% BSA in PBS was added and incubated for 2 h at RT. The samples were rinsed with PBS, and mounting medium with DAPI was added. The slides were viewed by BD pathway™ confocal microscopy (BD BioSciences, San Jose, CA, USA).

### 4.7. Immunohistochemistry (IHC) of β-Catenin and Collagen Type X

The immunohistochemistry staining of β-catenin and Collagen type X was performed using 5 μm sections. The sections were pre-incubated with 5 μg/mL proteinase K (Sigma Aldrich) for 10 min followed by 1 mg/mL hyaluronidase (Sigma Aldrich) for 40 min at 37 °C. Rabbit anti-human β-catenin antibody (Lifespan Biosciences, Seattle, WA, USA) or mouse anti-human Collagen type X antibody (BIOCYC GmbH & Co. KG, Cat. No. 2031501005, Brandenburg, Germany) was diluted 1:100 in PBS and incubated overnight at 4 °C. Non-immune controls underwent the same procedure without primary antibody incubation. The biotinylated secondary antibody was diluted 1:500 in 5% BSA in PBS and incubated for 30 min at RT. horseradish peroxidase (HRP)-Streptavidin was added and incubated for 30 min at RT. A DAB substrate kit (ab64238, Abcam) was used for visualization. The cell nucleus was counterstained with hematoxylin for Collage type X detection. Images were taken by using a Nanozoomer (Iwata City, Japan).

### 4.8. TUNEL Assay

Apoptosis of chondrocytes was detected using The DeadEnd^TM^ fluorometric TUNEL System (Promega). Nuclei were counterstained with Hoechst 33342. Images were viewed by means of BD pathway confocal microscopy.

### 4.9. GAG and DNA Assay

After 5 weeks of chondrogenic differentiation, the pellets were predigested in 250 µL of Tris-HCl buffer (0.05 M Tris, 1 mM CaCl2, pH 8.0) with 1 mg/mL proteinase K (Roche) for 16 h at 56 °C. Diluted samples (25 μL) were mixed with 150 μL 1,9-dimethylmethylene blue (DMMB) dye solution (3.04 g/L glycine, 2.38 g/L NaCl, and 20 mg/L DMMB in distilled water) and absorbance was measured at 525 nm. The relative cell number was determined by quantification of the total DNA using a QuantiFluor^®^ dsDNA System kit (Promega) according to the manufacturer’s instructions.

### 4.10. Enzyme Linked Immunosorbent Assay (ELISA)

The culture medium for pellet samples was collected weekly. The protein concentration of DKK1 was measured using ELISA according to the manufacturer’s guidelines (DKK1, R&D systems, Minneapolis, MN, USA). Secreted MMP1 in medium was also measured by ELISA using mouse anti-human MMP1 antibody (MAB901-SP, R&D systems), which was followed by incubation with a rabbit anti-mouse antibody coupled to a peroxidase. The amount of HRP was developed by the addition of Tetramethylbenzidine (TMB, 1-Step Ultra TMB-ELISA, Thermo Fisher Scientific). The reactions were stopped by the addition of H_2_SO_4_ and measured at 450 nm of the microplate reader.

### 4.11. Statistical Analysis

For the experiments using human bone marrow, samples were obtained from three donors. Each experiment was performed in triplicate. Data is expressed as the mean ± SD and analyzed by two-tailed student’s *t*-tests or one-way ANOVA. *p* < 0.05 was considered statistically significant.
